# The intelligent neonatal healthcare: a systematic review of machine learning architectures integrating the internet of medical things and blockchain

**DOI:** 10.3389/frai.2026.1802559

**Published:** 2026-04-21

**Authors:** Sarlinraj Madhalaimuthu, R. Sujatha

**Affiliations:** School of Computer Science Engineering and Information Systems, Vellore Institute of Technology, Vellore, Tamilnadu, India

**Keywords:** blockchain, intelligent healthcare systems, Internet of Medical Things (IoMT), Machine Learning (ML), neonatal healthcare, neonatal intensive care unit

## Abstract

**Background and motivation:**

Neonatal healthcare involves managing extreme physiological vulnerability, rapid disease progression, and time-critical decision-making within Neonatal Intensive Care Units (NICUs). Recent developments in blockchain, the Internet of Medical Things (IoMT), and Machine Learning (ML) have provided new opportunities of enhanced health-data governance, non-stop physiological tracking, and prevention of risks. Although this has been achieved, the past studies have primarily evaluated these technologies separately or with regard to adult health care conditions with little consideration of their combined relevance to neonatal care.

**Methods:**

In this study, a PRISMA-guided systematic review was conducted to examine intelligent neonatal healthcare systems that integrate ML, IoMT, and blockchain technologies. A systematic search of major scientific databases identified 122 records, of which 76 studies satisfied predefined inclusion criteria and were included for qualitative synthesis. The selected studies were discussed according to clinical areas of application, system architecture, evaluation practices, and implementation limitations peculiar to neonatal contexts.

**Synthesis of current evidence and identified research gaps:**

The review indicates that ML-based approaches are the most mature, particularly for early disease detection, mortality risk prediction, and clinical decision support. Continuous and remote physiological monitoring is the primary use of IoMT-based systems, but blockchain-based solutions are still mainly conceptual or prototype-based systems with primary concerns on data integrity, access control and trust. There are still fewer fully integrated ML–IoMT–Blockchain systems specifically for neonates, and there are persistent problems with interoperability, scalability, clinical validation, and AI lifecycle governance. Taking everything considered, this review combines disparate information, identifies important research gaps, and describes future research routes in line with Sustainable Development Goal 3 toward secure, trustworthy, and therapeutically useful intelligent infant healthcare systems.

## 1 Introduction

Artificial intelligence (AI) is the general ability of systems of computers to imitate human decision-making (Helm et al., 2020). Within this landscape, Machine Learning (ML) has taken on an especially consequential role, largely because it shifts the analytical burden from pre-specified rules to patterns learned directly from data-patterns that clinicians may not articulate explicitly yet rely on intuitively in practice. As emphasized in (Shah et al., 2023), ML-based methods hold substantial promise for deepening insight into disease trajectories and for tailoring clinical interventions in neonatal and pediatric populations in which early pathological signals are often faint, heterogeneous, and easily overlooked in routine practice.

The Internet of Medical Things (IoMT) extends these capabilities into the clinical environment by linking sensors, monitors, and smart devices into a connected infrastructure for neonatal care (Ashfaq et al., 2022). In practical terms, this ecosystem already includes applications such as smart inhalers and insulin pens, continuous oxygen saturation and cardiorespiratory monitors, and real-time biochemical sensing devices that enable remote and continuous follow-up outside traditional bedside encounters (Zhang et al., 2022). Rather than functioning as isolated tools, these devices contribute to a more granular and dynamic picture of the infant’s physiological state over time.

However, as data streams in neonatal care become denser, more continuous, and increasingly shared across institutional and even geographic boundaries, long-standing concerns about privacy, security, and trust acquire a new level of urgency. It is no longer sufficient to treat data protection as an afterthought or a purely technical add-on. Safeguarding the confidentiality of neonatal health records, specifying with precision who is permitted to access which subset of data, and enforcing responsibility when something goes wrong all demand coordinated efforts among clinicians, hospital administrators, technology developers, and regulatory authorities (Meisami et al., 2023). Beyond data security, blockchain infrastructures play a crucial role throughout the artificial intelligence lifecycle by enabling verifiable data provenance, supporting transparent model training and validation processes, and facilitating auditing and accountability of AI-driven medical decisions in neonatal care systems.

**Figure 1:**
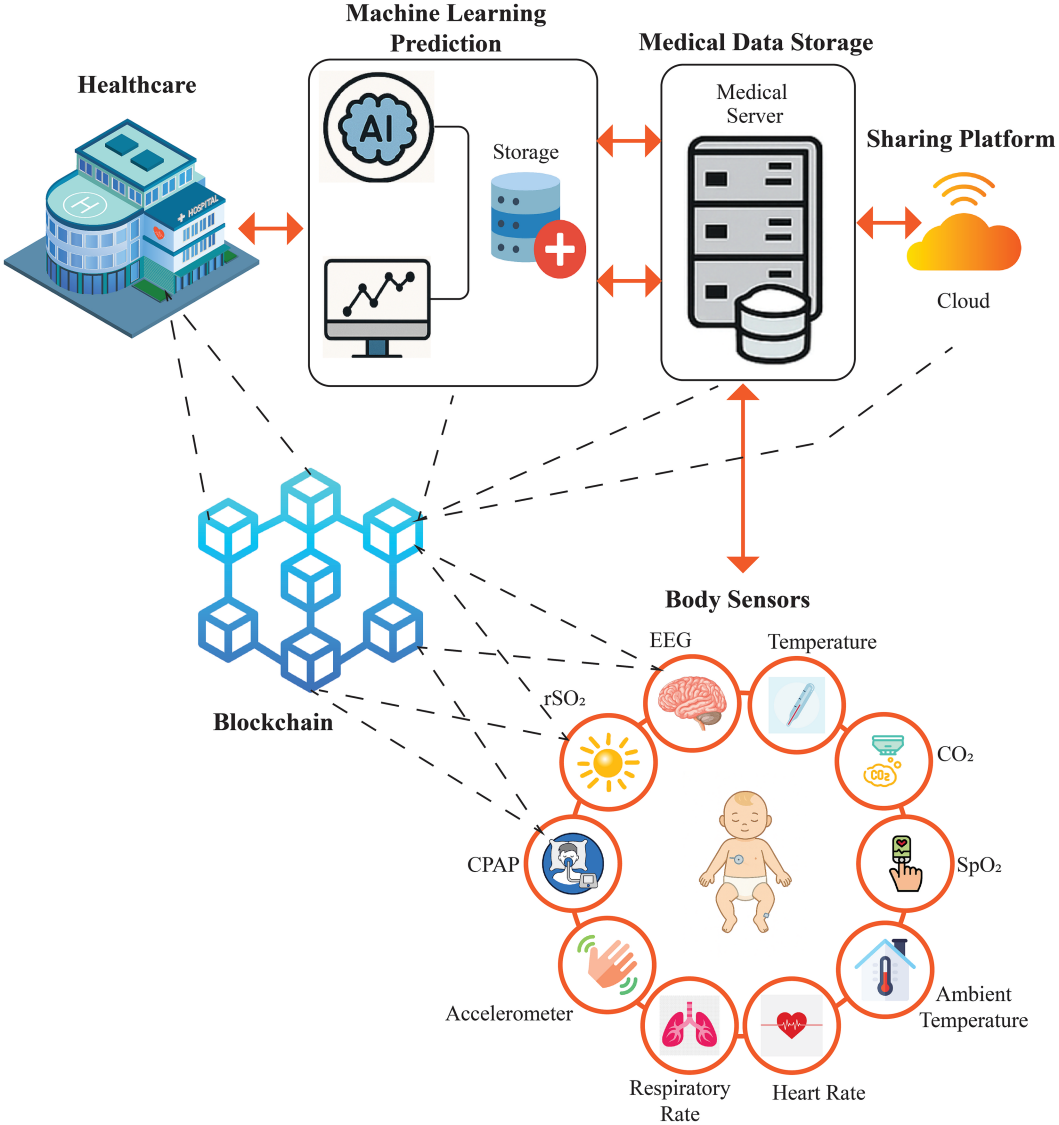
Conceptual architecture of an integrated Machine Learning (ML)–Internet of Medical Things (IoMT)–Blockchain framework for neonatal healthcare (icons adapted with permission from FreePik, Flaticon, Vecteezy, Dreamstime, Shutterstock, citypng, istockphoto, Clipart Library and iconsdb; Modern 3d urban hospital building with helipad on the roof isometric isolated vector illustration by macrovector, Stock Market Vector Icon 366110 Vector Art at Vecteezy by Muhammad Khaleeq, Database - Free medical icons by Smashicons, Blockchain - Free security icons by srip, Black line icon for database 42725622 Vector Art at Vecteezy by webtechops llp, Cloud Network, Wifi Zone Icon. Orange Version Stock Vector - Illustration of flat, connect: 2060758… by Hironicons, Clear Depiction Human Brain Anatomy Featuring Stock Vector (Royalty Free) 2684608195 | Shutterstock by mrvect02, Motion sensor - Free technology icons by Freepik, Illustration of a thermometer | Free Vector by rawpixel.com, Healthy Humain Pink Lungs Respiratory Vector Icon | Citypng by citypng, Pulse oximeter - Free technology icons by photo3idea_studio, Co2 Sensor Color Icon Vector Illustration Color Stock Illustration - Download Image Now - Backgroun… by Pavel Sevryukov, Red medical heartbeat line vector heart shape graphic in health charity concept | Free Vector by rawpixel.com, https://clipart-library.com/images/8czn5BL9i.png by Clipart Library, Cpap Therapy Sleep Apnea Insomnia Thin Stock Vector (Royalty Free) 1855692679 | Shutterstock by SilenceVideo, Room temperature - Free weather icons by Freepik, as well as ChatGPT AI image generation).

Figure 1 illustrates a unified neonatal healthcare ecosystem integrating Machine Learning (ML), the Internet of Medical Things (IoMT), and blockchain technologies. Within this context, the sensing provided by IoMT can sustain real-time and continuous data collection, clinical decision support and risk prediction with the help of ML models, and blockchain processes allow maintaining data integrity, access control, and accountability among stakeholders. It is in this integrated approach to framing the ecosystem that it makes sense that such a synthesis is required: to bring in the evidence of studies that examine such technologies together, not individually, into the context of neonatal care.

Therefore, this paper provides a synthesis of and a critical review of the literature on neonatal healthcare systems that unites the architectures of Machine Learning (ML) with Internet of Medical Things (IoMT) and blockchain-based governance. This review has taken an integrated approach of neonatal systems in examining how predictive intelligence, connected sensing and trusted data governance can work together to support intelligent neonatal care, unlike previous reviews that have mostly investigated these technologies individually. Besides the evidence base consolidation, the review finds a variation in the maturity of the three technological layers and proposes a multi-layer evaluation and benchmarking model specific to NICU limitations namely, predictive accuracy, sensing reliability, data governance, latency, interoperability, and deployment readiness. The study thus is based on a PRISMA-directed systematic review methodology and, besides providing a synthesis of the current evidence, it also provides a regulated basis to the design and evaluation of secure, scalable, and clinically actionable neonatal healthcare systems.

## 2 Methods

### 2.1 Review design and protocol

This paper used a PRISMA (Preferred Reporting Items for Systematic Reviews and Meta Analysis) based systematic review methodology to summarize the evidence on the health care system of neonatal intensive care that incorporates Machine Learning (ML), the Internet of Medical Things (IoMT) and blockchain technologies. PRISMA was adhered to enhance the transparency, reproducibility and rigor of the methods used in the identification, screening process, eligibility assessment, and inclusion.

**Figure 2:**
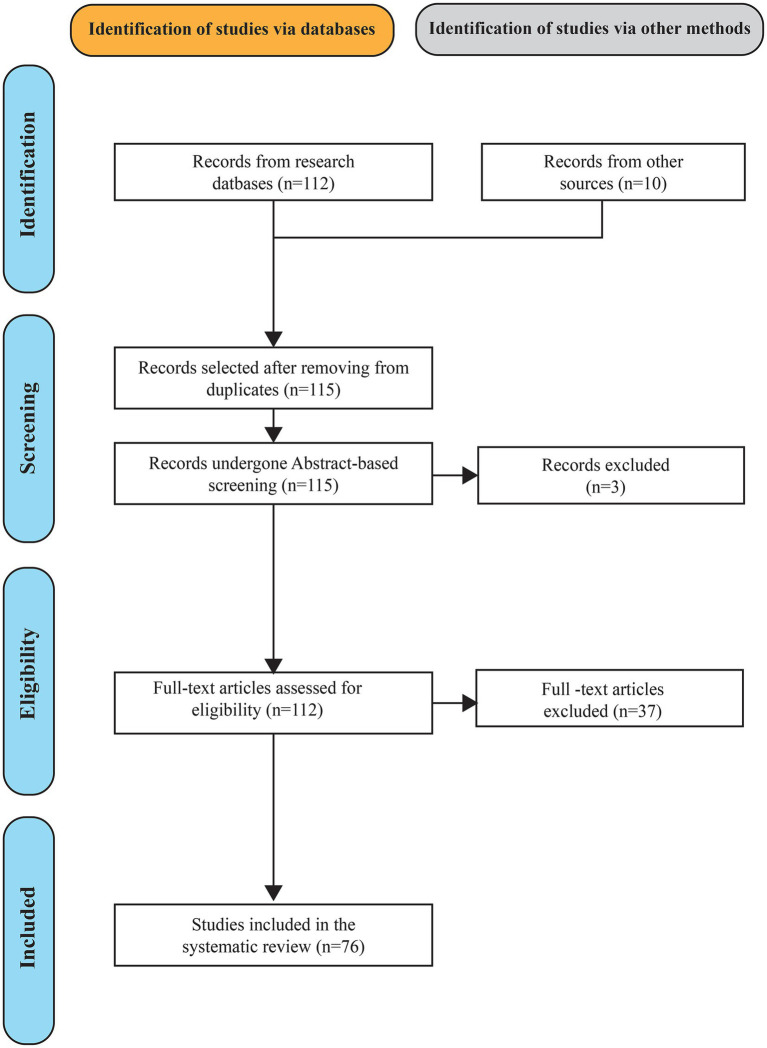
PRISMA flow diagram illustrating the study identification, screening, eligibility assessment, and inclusion process.

The review protocol was comprised: (i) specification of the research questions, (ii) searching the records in the existing scientific databases in a systematic manner, (iii) inclusion and exclusion criteria were used in screening of records in title and abstract and full texts, and (iv) synthesizing the eligible studies were qualified in a qualitative manner. Figure 2 presents the PRISMA steps (identification, screening, eligibility, and inclusion) of the study selection procedure as a summary.

### 2.2 Research questions

This review is based on a number of predetermined research questions (RQs) in order to explore the integration of Machine Learning (ML), the Internet of Medical Things (IoMT), and blockchain technologies in neonatal healthcare systematically. The RQs are designed to make the evidence synthesis operationalized through data extraction and its comparison across the studies to include end to end architecture and data flow features, application domains of the clinical area, trends in methodologies related to the learning and sensing pipelines and evaluation and performance reporting patterns, and the technical, clinical, and governance phenomena that have not been resolved to initiate deployable intelligent neonatal care systems.

### 2.3 Data sources and searching strategy

**Figure 3:**
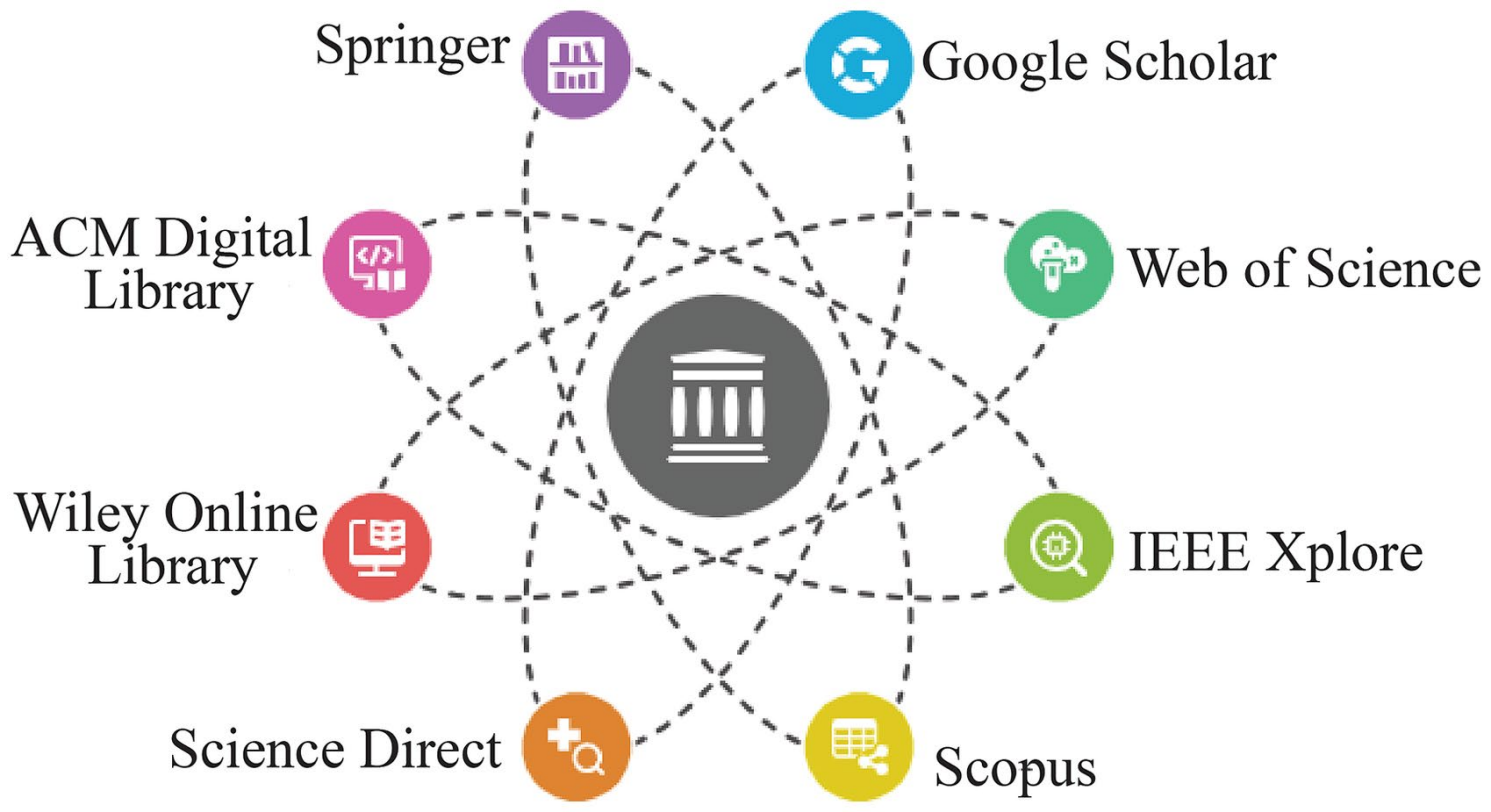
Databases and information sources used for literature retrieval in the PRISMA-guided systematic review of ML–IoMT–Blockchain applications in neonatal healthcare (icons adapted with permission from Flaticon, Freepik, iconsdb, Vecteezy and Wikimedia Commons; Letter g - Free education icons by Md Tanvirul Haque, Computer icon | Premium Vector by Vectoristock, Deep pink code optimization 2 icon - Free deep pink seo icons by iconsdb, Gears Line Shadow Circle Icon Design 43895225 Vector Art at Vecteezy by Muhammad Usman, Sharethis - Free social media icons by Freepik, Table Generic gradient outline icon | Freepik by Iconshadow91and File:Resources icon.png - Wikimedia Commons by EducIcons under CC-BY SA 4.0).

To answer the review questions presented in Table 1, a systematic literature search has been conducted in large bibliographic databases, digital libraries, and academic search services such as Web of Sciences, Scopus, IEEE Xplore, ACM Digital Library, ScienceDirect, SpringerLink, Wiley Online Library, and Google ScholarA systematic literature search was conducted for studies published from database inception to 2025. The search strategy was constructed on the basis of four concept areas, which included neonatal healthcare, machine learning/artificial intelligence, Internet of Medical Things (IoMT)/IoT-enabled monitoring, and blockchain-based security and governance. Synonymous words in each of the concept groups were linked together with the Boolean operator OR and the four concept groups were linked together with the Boolean operator AND. The researchers used a standard information sources mentioned in the following Figure 3.

**Table 1:** Research questions guiding the systematic review of ML–IoMT–Blockchain integration in neonatal healthcare.

RQ. No	Research questions	Motivation
1	What are the unsaturated issues that exist in neonatal healthcare?	The answer to this question lists the unexplored challenges that exist in the field of neonatology. The unresolved issues in the field of neonatology are laid out in the answer to this question.
2	What ML applications are developed to resolve the neonatal issues?	The answer to this question analyses the innovative solutions and clinical workflows developed for better neonatal healthcare outcomes.
3	What are the neonatal healthcare applications developed using ML and the IoMT?	The answer to this question outlines the innovations developed for neonatal healthcare using the two cutting-edge technologies ML and IoMT.
4	What are the neonatal healthcare applications developed using Blockchain and the IoMT?	The answer to this question brings out the secure, efficient, and patient-centric neonatal healthcare systems designed using Blockchain and IoMT.
5	How can ML, the IoMT and Blockchain be integrated to solve neonatal healthcare issues?	The answer to this question explores the comprehensive frameworks that are developed for neonatal health care using ML, Blockchain, and IoMT.
6	What future directions and opportunities are identified for ML, IoMT, and blockchain in neonatal healthcare?	The answer narrates the future innovations and growth possibilities of the technologies ML, BC, and IoMT.
7	Research gaps in the integration of ML–IoMT–Blockchain for neonatal healthcare	The answer gives unified digital framework and co design of tri layer architecture to meet clinical requirements in NICU environments.

### 2.4 Inclusion and exclusion measures

Our search outcomes are refined through a set of exclusion criteria (EC) and inclusion criteria (IC).

#### 2.4.1 Inclusion measures

- The research articles considered for the work should be indexed in the standard research databases.
- The scientific publication should be written in English and indexed by SCOPUS, WoS, and other standards.
- The study must apply ML, IoMT, or blockchain within healthcare, with explicit relevance to neonatal or perinatal contexts.
- Research papers that address the ideas of “Big Data in Healthcare”, IoMT, and Blockchain for medical settings.
- Applications of ML, IoMT, and Blockchain developed for the healthcare domain are covered in the study.
- The article describes how Blockchain technology is integrated with ML and IoMT.
- The answers to the research questions ought to be included in the paper.

#### 2.4.2 Exclusion measures

- If the same article is available more than once, it can be removed.
- The article is not prepared by following a certain standard research framework.
- Research works that do not cover the “Blockchain technology, IoMT, and ML” technologies.

### 2.5 PRISMA compliance

This systematic review was conducted and presented in line with the Preferred Reporting Items for Systematic Reviews and Meta-analyses (PRISMA 2020) model. The review workflow was organized with the help of PRISMA guidelines, which presumes the study identification, the exclusion of duplicated materials, the screening of titles and abstracts, the eligibility screening based on the full-text, and the inclusion of the relevant studies. Figure 2 summarized the PRISMA-based screening and selection pathway which was employed in this review. PRISMA was used in order to enhance transparency, consistency, and reproducibility of the review process methods.

### 2.6 Study selection process (PRISMA)

Study selection had been carried out in accordance with the PRISMA strategy to provide an open and repeatable screening process. As shown in Figure 2, the study was selected by doing identification, screening, eligibility, and ultimately, inclusion. In the identification, 122 records were obtained in scientific databases and other sources. Following the removal of duplicates, the number of distinct records to be screened was 115. Relevance with regard to neonatal healthcare and the unified implementation of Machine Learning (ML), the Internet of Medical Things (IoMT), and blockchain was then filtered on titles and abstracts, putting it down to 112 reports to be reviewed in full.

During the eligibility phase, 112 full-text reports were assessed against the predefined inclusion and exclusion criteria. A total of 37 reports were excluded due to lack of relevance to neonatal healthcare, insufficient rigor, and inadequate reporting quality. 76 studies met all the eligibility requirements and were included for qualitative synthesis. The studies included were then classified according to the area of application and analysed to respond to the review research questions.

## 3 Results

### 3.1 Challenges addressed in neonatal healthcare

Persistent challenges in neonatal healthcare are the areas that need specific clinical and systemic advancement. Based on recent research and expert opinions, some of the persistent challenges in neonatal healthcare are listed in the following Table 2.

**Table 2:** Key clinical and system-level challenges in neonatal healthcare identified in the literature.

Author/Reference	Summary of identified issue
Fanaroff and Fanaroff (2020)	High burden of neonatal infections and limited capacity for early detection.
Kampalath et al. (2022)	Inadequate maternal, newborn, and child health services in conflict settings, lack of high-quality care.
Ward and Beachy (2003)	Complication associated with preterm birth remains a major cause of neonatal morbidity and mortality.
Getaneh et al. (2022)	Persistently high neonatal mortality rate, Identifying and addressing the root causes is crucial.
Bolisetty et al. (2014)	Inadequate nutrition, feeding difficulties for neonates.
Hadders-Algra (2021)	Neurodevelopmental disorders with insufficient early detection and the interventions.
Rahman et al. (2023)	Low quality of healthcare delivery – Low rate of breastfeeding rates, High rates of death due to asphyxia in Special Newborn Care Units (SNCUs)
Tinker et al. (2005)	9% of newborns require specialized care – Lack of medical facilities and trained experts.
Temur et al. (2025)	High prevalence of premature birth, lack of Unified Medical Strategies, and Need for Public Health Initiatives

### 3.2 ML-based neonatal prediction and decision-support applications

Numerous applications aimed at solving many of the ongoing clinical issues related to newborn healthcare have been built as a result of machine learning. These applications support early diagnosis, physiological monitoring, mortality prediction, resuscitation assistance, and clinical decision-making. Representative examples from the literature are summarized below.

#### 3.2.1 ML for early diagnosis of neonatal diseases

Several studies developed ML-based diagnostic system targeting conditions such as sepsis, respiratory distress syndrome, perinatal asphyxia, and infections.

A knowledge-based system for detecting neonatal diseases applying C Language Integrated Production System (CLIPS) is developed where ML merges with expert knowledge to identify sepsis, respiratory distress syndrome, and perinatal asphyxia. By aligning with the Sustainable Development Goals to decrease neonatal mortality rates, the PART (Rule-based categorization algorithm) achieved accuracy of 98.06% in neonatal disease classification (Wendimu and Biredagn, 2023). A multivariate regression model based on deep neural networks (DNNs) was developed, and it was validated by different ML algorithms, like K-Nearest Neighbors (KNN), Support Vector Machine (SVM) to accurately predict the in-hospital mortality of neonates who had clinically suspected sepsis. The DNN achieved the highest accuracy of 95.64% and AUC of 0.923 (Hsu et al., 2021).

López-Martínez et al. developed a neural network model, which served as a screening tool for early neonatal sepsis prediction, providing decision support for timely antibiotic administration, and achieved 80.32% sensitivity, 90.4% specificity, and 83.1% precision with a 92.5% AUC on a test dataset (López-Martínez et al., 2019). The SHapley Additive exPlanation (SHAP) method and supervised machine learning techniques SVM, XGBoost, Logistic Regression, and Random Forests are used on the SPNeoDeath dataset (15,858 records) for informed decision-making in reducing neonatal mortality and achieved an average accuracy of 0.96 (Beluzo et al., 2020). Robi and Sitote utilized Asella Comprehensive Hospital (2018–2021) data, built a classification stacking model, and achieved 97.04% correctness in the early diagnosis of resource-limited health facilities (Robi and Sitote, 2023).

Collectively, these studies indicate that ML-based diagnostic models are predominantly applied to early sepsis detection and mortality risk assessment in neonatal populations.

#### 3.2.2 ML using imaging and sensor data

Machine learning models have been applied to thermal imaging, chest movement sensors, and video data. Ornek et al. employed deep convolutional neural networks (CNNs) and infrared thermography (IRT) to determine the health state of newborns. Thermal images from a NICU were augmented to create a dataset for CNN training, offered the possibilities for earlier disease detection and reduced mortality, and achieved a high Area Under the Curve (AUC) of 99.58% in neonatal image classification (Ornek et al., 2019). Munz and Wolf introduced a methodology for classifying breathing patterns using a thorax sensor to prevent sudden infant death (SID) incidents. A mechanical thorax simulator generated the sensor data for various breathing patterns, supported the development of a wearable chest belt sensor to monitor infant breathing, and predicted the SID incidents through ML algorithms (Munz and Wolf, 2019).

Infrared thermal imaging is a valuable tool for neonatal research, which offers advantages in monitoring thermoregulation and diseases like necrotizing enterocolitis. Infrared thermal imaging is used to measure the physical temperature of the neonates without physical contact and enhance clinical surveillance, potentially reducing medical costs and hospital (Knobel et al., 2011). To recognize activity in low-quality video recordings of infant resuscitation, the scientists suggested a two-step deep neural network system called ORAA-net. This system uses Convolutional Neural Networks (CNNs) and 3D CNNs. With its excellent precision, recall, and accuracy, the ORAA-net system demonstrated a method to increase training and quality improvement in infant resuscitation in Tanzania by optimizing treatment guidelines (Meinich-Bache et al., 2020).

Nguyen et al. provided a low power AI-assisted real-time sonification system that can be deployed to continuously track the position of the neonatal seizures in the NICUs. The system can transform multichannel EEG into an adaptive audio that reveals areas that are inclined to seizures through an integration of DSP and ML-based seizure probability detection. It is executed on an AI microcontroller and optimizes processing time to the lowest and improves clinical responsiveness compared to past offline or power-hungry designs (Nguyen et al., 2025).

#### 3.2.3 Mortality and morbidity prediction models

A substantial number of ML applications support prediction of neonatal mortality, neurodevelopmental (NDI), and early deterioration.

A retrospective review of 5 years’ worth of neonatal records was utilized by Iqbal et al. to construct the logistic regression model which predicted sepsis mortality and potentially improved the treatment decisions and outcomes in NICUs using the WEKA tool. The model achieved 88.4% accuracy and an ROC (Receiver Operating Characteristic) of 0.906 using the OneR attribute evaluation + Ranker method (Iqbal et al., 2023). With an AUC ranging from 0.80 to 0.874, a retrospective study sought to create an ML model that could predict neonatal sepsis in 4 h before clinical identification using data from electronic health records (EHRs) (Masino et al., 2019). Using the data from 229 neonates in the NICU, Shirwaikar et al. employed decision trees, SVM, and random forest, for predicting neonatal apnoea, focusing on preterm infants with an accuracy: of 0.88, kappa (Cohen’s kappa (*κ*) statistic): 0.72 (Shirwaikar et al., 2015). Neonatal Mortality Risk Assessment research proposed a novel model using computer vision techniques, custom convolutional neural network architecture, and machine learning classification. This method assessed the risk of neonatal death by using the public health data from São Paulo and achieved an accuracy of 90.61% in death detection (Beluzo et al., 2020).

To create ML models that might anticipate neonatal fatalities in NICUs, researchers used algorithms such as ANN, decision trees, SVM, and Bayesian network and achieved the greatest AUC (0.982 and F-score) (Sheikhtaheri et al., 2021). Linear Discriminant Analysis (LDA) was done by Kefi et al. to predict short-term mortality in NICU based on data from the first 2 h of admission. Relevant features were selected, various classifiers were tested and the approach yielded promising results with high accuracy in the neonatal domain (Kefi et al., 2019). Using an artificial neural network (ANN) and a logistic regression model, 890 neonates, born between 1990 and 1993 were tested. The work attempted to forecast the risk of newborn mortality for preterm infants. It attained an area under the receiver operator curve of 0.95 opposed to 0.92 (Zernikow et al., 1998).

The BAG model (Birth weight, Apgar score at 5 min of Gestational age) is a mortality prediction model for neonates developed by Moreira et al. using Swedish Neonatal Quality Register data with an AUC of 76.9% in the development cohort and 68.9% in the validation cohort (Moreira et al., 2022). Sequential and logistic regression were used as predictive models of death in premature infants born at ≤30 weeks gestation or with a birth weight of ≤1,500 g in eight new models created between June 2010 and July 2019, mostly in North America and Europe (del Río et al., 2020). Gera and Ramji found birth weight under 1,200 g to be an equally effective predictor as the CRIB score, suggested that VLBW neonates with cardio-pulmonary disturbances, especially those who need mechanical ventilation, have a high risk of early neonatal mortality (Gera and Ramji, 2001). The Italian Neonatal Network’s 23,747 neonates’ data is analyzed using the Preterm Infants Survival Assessment (PISA) predictor, an ML technique, designed to forecast the survival rates in preterm infants. This helps to improve risk adjustment and results in comparisons among institutions in the neonatology sector (Podda et al., 2018).

Growth for age, sex, and culture, the absence of congenital malformations, antenatal steroid use, Apgar score, admission temperature, and respiratory status are important predictors of survival beyond gestational age and birth weight in the research (Medlock et al., 2011). Multivariate models were developed for infants with uncertain prognoses based solely on age and weight. According to the article de Castro et al. low Apgar scores at 5 min, male gender, low birth weights under 1,000 g, and inadequate hospital facilities are all associated with a significant rate of neonatal mortality within the first 24 h. To lower the rate of early infant mortality in Northeast Brazil, the study suggested improving hospital facilities and obstetric treatment (de Castro et al., 2016). Ambalavanan et al. used classification tree analysis on Trial of Indomethacin Prophylaxis in Preterm (TIPP) data, including gestational age, birth weight, fluid intake, and transfusion needs, to predict the mortality or neurodevelopmental impairment (NDI) in premature babies (Ambalavanan et al., 2006).

#### 3.2.4 ML for growth and nutritional support

Extreme Gradient Boosting (XGB) was used to predict Postnatal Growth Failure (PGF) in low birth weight infants to optimize nutritional support (Han et al., 2022).

#### 3.2.5 ML for decision support and family-centered NICU care

Physician-Parent Decision Support System (PPADS) for family-centered care in NICUs was developed to provide clinical updates to neonatologists and decision-making support to parents (Frize et al., 2013). The Physician-Parent Decision Support (PPADS) tool, which includes modules for parents and doctors to help with neonatal care decisions for infants, is used to evaluate the mortality risk. Beyond clinical expectations, the development of the open-source ANN-Builder handled real-time data and automated mortality risk assessments, enabling real-time result forecasts with plans for future alarms on significant changes in risk predictions (Frize et al., 2015).

Townsend and Frize explored the application of ANNs Clinical Decision Support (CDS) systems that predict the survival level, mortality, Number of days, and period of intubation in NICUs by utilizing data from the Canadian Neonatal Network (Townsend and Frize, 2008). With the aid of C5.0 decision tree software, newborn mortality prediction models are created utilizing real-time medical measurements. The study found that serum pH and mean blood pressure, measured within the first 48 h of NICU admission were significant predictors of mortality. The proposed a top-performing model, demonstrated potential therapeutic utility with over 60% sensitivity, 90% specificity, and a positive predictive value of 38% (Gilchrist et al., 2011).

#### 3.2.6 ML-assisted neonatal resuscitation

The experiment showed that it is possible to use AI to identify activities in neonatal resuscitation with the help of video recordings. Anticipated impacts using scientific advancements in AI, improved medical training, guidelines, and potentially better outcomes for newborns requiring resuscitation (Engan et al., 2023). Hsu, Yang, et al. explored the random forest (RF) model, to predict mortality in neonates undergoing mechanical intubation for respiratory failure with superior accuracy compared to traditional scoring systems. The RF model’s prediction emphasized the potential of ML techniques that can improve healthcare solutions in the NICU setting (Hsu et al., 2021). Urdal et al. addressed the difficulties associated with early neonatal death, with an emphasis on detecting the stimulating deeds during newborn recovery using ECG and accelerometer information. The suggested approach, NBstim, which used the NeoBeat gadget, was 90.3% accurate overall and significantly reduced neonatal mortality (Urdal et al., 2020).

The study by Rettedal et al. evaluated the effects of continuous and instantaneous heart rate (HR) feedback on the timing of the start of positive pressure ventilation (PPV) and the short-term results in neonates following delivery utilizing a dry electrode ECG sensor. The usage of this sensor for continuous HR monitoring just after birth was compared to standard HR assessment methods in this randomized controlled trial. The major goal of the research was to determine what percentage of babies received PPV within 60 s (Rettedal et al., 2022).

A 2-stage classifier trained on data from 30 resuscitated neonates was used to classify the recovery activities for birth-asphyxiated newborns using acceleration and ECG signals. An average accuracy of 79% was attained in the classification (Vu et al., 2017). In low- and middle-income nations in particular, Bettinger et al. emphasized the use of evidence-based resuscitation techniques to reduce infant mortality from birth-related respiratory depression. To improve adherence to resuscitation protocols, it examined learning methodologies and concentrated on clinical decision support and post-event reflection (Bettinger et al., 2021).

Overall, the literature analysis of the ML studies demonstrates that the neonatal machine learning research is the most advanced in sepsis, mortality prediction, and decision support. Growth monitoring, family-centered care as well as real-time integrated deployment, in contrast, are relatively under explored. In these studies, retrospective modeling is the most utilized whereas externally validated and clinically deployed ML systems are few.

### 3.3 IoMT enabled neonatal monitoring and sensing system

Das et al. created a clever e-healthcare system that uses machine learning to forecast hospital NICU unit availability. The system achieved good recall, precision, and accuracy rates by combining models from Convolutional Neural Network (CNN) and Support Vector Machine (SVM) (Das et al., 2023). Recent studies reported the use of ML-enabled IoMT systems to support neonatal monitoring, physiological data aggregation, and resource management in healthcare environments (Shirwaikar et al., 2015; Matsushita et al., 2022). Table 3 summarizes representative neonatal healthcare application developed using ML and IoMT. These articles also emphasized the value of supervised machine-learning techniques for monitoring and forecasting neonatal illnesses. Although some included studies primarily focus on maternal health monitoring, they were retained due to their architectural relevance and direct applicability to neonatal IoMT sensing and ML-based risk prediction pipelines.

**Table 3:** Machine Learning–enabled Internet of Medical Things (IoMT) applications for neonatal and neonatal-relevant monitoring, sensing, and operational support.

Author	Application	Key contribution
Das et al. (2023)	Smart e-Healthcare System for NICU Units	A smart e-healthcare structure is created to calculate the readiness of the adjacent NICU units.
Nachabe et al. (2015)	A Mobile Health application called as Neonatal Incubator Monitoring System (NIMS) using IoMT and Co-AP protocols.	NIMS can accumulate and distribute biomedical data of early born infants in an incubator. The medical staff and parents can access the data. The paper also integrates the system with IoT techniques using CoAP protocol and URIs.
Zarehpour et al. (2023)	BiliBin: An Intelligent Mobile Phone-based Platform to Monitor Newborn Jaundice	The model involved using smartphone cameras to capture images of the skin of a newborn’s sternum. Bilirubin levels are estimated using machine learning regression models in these images, allowing for a non-invasive effect for blood tests.
Munyao et al. (2024)	Real-time pre-eclampsia prediction model	This was wearable, pre-eclampsia watch that included biosensors that will monitor the vital signs of pregnant women and transfer this information to a cloud-based system via LoRa. Naïve Bayes was found to be better at predicting the risk of pre-eclampsia compared to other machine learning models. The system generated real-time alerts to help prompt intervention to improve outcomes.
Fouad et al. (2020)	Maternal health risk prediction using machine learning and internet of medical things	The study used ML classifiers and IoMT sensors (heart rate, BP, and temperature) to process continuous physiological measurements in an attempt to identify pregnancy-related risks early. IoT devices collect real-time maternal health indicators, which form the input for ML models that detect disorders and facilitate appropriate action. Although this does not imply a specific ML to IoMT synergy for the newborn, this framework suggests that it is directly applicable to the newborn.

These studies report the application of IoMT-based sensing systems combined with ML techniques for neonatal monitoring, physiological data collection, and operational support.

### 3.4 Blockchain supported neonatal monitoring, records, and trust mechanism

A limited number of researches are done by integrating Blockchain and IoMT which are briefly summarized in Table 4.

**Table 4:** Blockchain-enabled Internet of Medical Things (IoMT) applications for neonatal healthcare monitoring, data management, and trust mechanisms.

Author	Application	Key contribution
Madhusudhan and Pravisha (2023)	Blockchain-Enabled IoMT for NICU Infant Health Monitoring System	The system was designed to manage NICU data—the parameters of prematured newborn babies in real-time, securely, and scalable. The data is divided as critical and non-critical using deep learning network based on CNN. Fog used for the instantaneous analysis of critical data. It employed a private Blockchain to store important data both permanently at the decentralized cloud layer and temporarily at the fog layer. The framework offers several advantages, such as reducing latency, enhancing data security, privacy, facilitating data analysis and improving patient outcomes.
Chauhan et al. (2021)	Smart cradle application to observe the new born infants based on IoMT and Blockchain	A smart cradle system for monitoring babies in new-born wards is developed using Blockchain and IoMT. It improved the security and comfort of newborns in hospital environments by utilizing a moisture sensor, thermal scanner, FSR sensor. The proposed solution can diminish the difficulties of the parents and healthcare staff, prevent infant abduction, detect wet nappies, monitor body temperature, and generate alarms and alerts in case of any abnormality.
Saxena et al. (2022)	Blockchain and IoMT-Integrated Healthcare Application, which contains Medchain-IoMT, HealthBlock, Blockcare	Underlining the security and privacy of the medical records system based on Blockchain and the monitoring of patients with the help of IoMT is the objective of this research. Blockchain can be used to follow the drugs in the supply chain and protect the data secured from the medical field. The paper acknowledged the limitations and challenges of implementing Blockchain and IoT technologies, such as resource constraints, scalability, interoperability, and standardization. The paper explored the potentials of these trends in healthcare applications, such as patient monitoring, drug traceability, and medical record management. Provided future research direction applicable to neonatal systems, including algorithms, protocols, ethical frameworks, and standardization.
Alex et al. (2024)	Conceptual Integration of Blockchain and Smart Healthcare.	Blockchain and IoMT integration in neonatal healthcare allows for secure and real-time monitoring of newborn vital signs via decentralized data exchange. It provides tamper-proof storage, improves interoperability among healthcare devices and platforms, resulting in better care coordination.
Babatunde et al. (2022)	Chain Code Blockchain-based Key Agreement Authentication Mechanism (CCBKAAM)	CCBKAAM integrated a signature method to authenticate IoMT things securely. It mainly combined IoT and Blockchain for the safety of medical things. This study relevant to neonatal environments where reliable and authenticated device interactions are critical for clinical safety.
Othman and Getahun (2025)	Blockchain + IoMT enabled EHR monitoring system	Introduced a secure, scalable architecture that links IoMT sensors to a permissioned blockchain for real-time patient monitoring and tamper-proof health-record management. This system detected abnormal sensor readings and logs them as blockchain transactions; supports flexible mapping of patients–sensor relationships. Overall, Provided strong performance: 150 ms encryption, 250 ms blockchain recording, 500 ms PoW consensus, 80 ms alert generation, and 60 ms DB retrieval, demonstrating suitability for real-time healthcare.

The reviewed studies primarily describe blockchain-enabled architectures for secure data handling, authentication, and record management in IoMT-based healthcare systems.

### 3.5 Integrated ML–IoMT–Blockchain frameworks in included studies

Few studies directly indicate the combination of the use of Machine Learning (ML), the Internet of Medical Things (IoMT) with blockchain technologies in one healthcare system. Table 5 summarizes selected literature that suggests or tests integrated ML–IoMT–Blockchain architectures applicable to healthcare systems, such as neonatal and neonatal-relevant ones.

**Table 5:** Representative studies proposing or evaluating integrated Machine Learning (ML)–Internet of Medical Things (IoMT)–Blockchain architectures applicable to healthcare, including neonatal and neonatal-relevant systems.

Author	Application	Key contribution
Krishna et al. (2025)	CovMedCare – Integrated model that includes IoMT, Blockchain, and ML	CovMedCare solution was applied to healthcare monitoring scenarios including neonatal contexts by combining IoT, machine learning and blockchain technologies. IoMT devices measured with the help of WBANs to detect the vital signs of neonatal patients in real-time. Machine learning examined this information when the health issues are still in their initial phases of detection. The blockchain technology permited safe and non-distorted storage and transfer of health records that contain sensitive data. A set of these tools supports early-stage detection and data-driven monitoring and improve the safety, effectiveness, and transparency of newborn care.
Khan et al. (2025)	ML–IoMT–Blockchain integrated architecture	The BDLT-IoMT framework proposed as a secure and scalable framework that integrates SVM-based machine learning for resource optimization and blockchain-enabled data integrity. This study demonstrated the potential for IoMT devices, machine learning algorithms, and distributed ledgers to work together through on-chain/off-chain communication, IPFS-based storage, and secure multi-node interoperability, thus describes an integrated architectural framework into integrated neonatal healthcare systems.
Wang et al. (2025)	Blockchain enabled framework for MIoT healthcare system	Combined Blockchain and collaborative learning to provide privacy and secure model training on distributed IoMT devices. Extra Trees Classifier (ETC) has the highest overall performance, as it demonstrated better Accuracy, Balanced Accuracy (BA), F-Measure (FM) and Matthews Correlation Coefficient (MCC) in the federated environment, compared to all other tested models (AdaBoost, Extra Trees, Decision Tree, and LDA). The framework has showed a scalable and insecure diversification of ML + IoMT + Blockchain, which provides a transferable structure that can be used in neonatal IoMT backgrounds to aims to support secure and reliable clinical forecasting.
Jain and Kumar (2025)	REDC: Resource efficiency driven consensus for healthcare IoT-Blockchain system	Presented a blockchain consensus protocol optimized to healthcare IoT devices with resource constraints and aided by machine learning. Bases on an e-greedy ML tuner and dual-hashing (SHA-256 + DLH) to minimize the energy consumption, latency and computational cost in running continuous medical equipment. This framework but it provides a scalable and secure framework that can be used by NICU IoMT sensors, which can be used to provide low-power real-time and tamper-proof neonatal monitoring.
Patni and Lee (2024)	EdgeGurard Blockchain secured learning and IoMT architecture for centralized medical resource orchestration	EdgesGuard proposed decentralized architecture that combines blockchain, IoMT, and collaborative learning to provide secure orchestration of medical resources over distributed healthcare networks. Introduced a featherweight blockchain consensus, reinforcement-learning-based sum of edge resources allocation, and differential-privacy FL to safeguard patient data, however, enhance training efficiency. Research Showed higher performance in computational efficiency, data integrity, privacy and scalability to various medical datasets, indicating the worth of ML–IoMT–Blockchain synergy to large-scale collaborative healthcare systems.
Bhasker et al. (2025)	Blockchain, IoMT sustainable healthcare system for disease diagnosis, intrusion detection, and proactive healthcare monitoring in IoMT network.	Research provided Blockchain, IoMT sensors and collaborative learning to establish a safe, adaptable, and smart healthcare environment. A high accuracy in diagnosing diseases (96.425%), its ability to detect intrusion (97.16%), and a high level of privacy (98.73), which reports performance metrics across ML, IoMT, and blockchain components of ML + IoMT + Blockchain across its end. An example of this is the fact that, although not neonatal, the framework can be easily extended to NICU contexts, including the use of safe neonatal sensor feeds, privacy-conscious model training, and clinical real-time monitoring.

In the articles in the sampled studies, integrated ML–IoMT-Blockchain devices are mainly described as theoretical frameworks or initial designs, and little neonatal-specific validation and heterogeneous assessment plans.

## 4 Discussion

### 4.1 IoMT adoption and system-level constraints in neonatal care

The existing literature of neonatal IoMT demonstrates impressive innovation in remote monitoring, sensor-based evaluation, and early risk detection, but remains fragmented across ML–IoMT implementations and security-focused approaches. Although the use of ML models and IoMT frameworks proves to be feasible in non-invasive neonatal monitoring, a lack of security with the use of Blockchain technology, a small scale of data, and in-depth clinical validation indicate significant gaps. To develop secure, scalable, and feasible newborn healthcare systems, future neonatal systems will require tighter integration of ML-driven analysis, IoMT sensing, and blockchain-powered data governance.

### 4.2 Blockchain maturity and governance challenges in neonatal systems

Through the literature, it is apparent that Blockchain-IoMT systems are moving toward secure and real-time neonatal monitoring, data integrity, authenticated interactions between the devices, and enhanced management of care. Although these solutions are enhancing privacy and operational reliability, the majority of them are on a prototype level and not integrated with ML-driven neonatal predictions or a large-scale clinical assessment. Continuing issues with scalability, interoperability, and small neonatal data sets indicate that a fully integrated system using machine learning, IoMT, and blockchain in neonatal care has yet to be developed. Therefore, substantial scope remains to achieve clinically validated, intelligent, and standardized neonatal healthcare next-generation architectures.

Nonetheless, not every neonatal healthcare function can be relevant to blockchain. It can not be appropriate in high-frequency, continuous, ultra-low-latency bedside decision loops, continuous high-frequency physiological data streams, and resource-constrained IoMT deployments, where the consensus overhead, replication of storage, and transaction delay can be used to decrease system responsiveness. Blockchain is not also suitable in the case when sensitive clinical data have to be changed, fixed, or erased by legal, ethical, or institutional government requirements. In these environments, more convenient options can include centralized databases or off-chain/on-chain versions, where blockchain will only be used to store audit trails, consent management, and access-control logs as opposed to actual real-time monitoring data.

### 4.3 Maturity of integrated ML–IoMT–Blockchain architectures

The analyzed articles all point to the fact that there is a rising trend toward using ML, IoMT, and Blockchain to improve security, prediction quality, and real-time surveillance of neonatal and health care systems in general. Although these hybrid structures enhance data integrity, device authentication and clinical decision-support, the majority of solutions are conceptual or general-purpose, as opposed to neonatal ones. Massive gaps exist in terms of interoperability, energy efficient architecture and clinical validation in the NICU setting. In such a way, comprehensively integrated ML solutions combining with IoMT and Blockchain neonatal solutions represent an emerging area of neonatal health informatics research. In such a way, comprehensively integrated ML solutions combining with IoMT and Blockchain neonatal solutions represent an emerging area of neonatal health informatics research.

Taken together, the literatures reviewed indicate that the predictive applications of ML in neonatal care are more developed, IoMT-based neonatal sensing systems are developing, but still fragmented, and blockchain-based governance is at an early stage of direct NICU application. Such an unequal maturity within the three levels justifies the necessity of integrated and clinically validated design of a neonatal system.

### 4.4 Proposed evaluation and benchmarking framework for neonatal ML–IoMT–Blockchain systems

Based on the synthesis of evaluation practices reported across the included studies, this review proposes a unified evaluation and benchmarking framework for ML–IoMT–Blockchain-based neonatal healthcare systems.

The suitability of neonatal healthcare of ML–IoMT–Blockchain systems is to be measured based on a multidimensional framework of measures to assess predictive performance, sensing reliability, security, governance, and end-to-end operational efficiency. In the case of ML components, performance is commonly evaluated in terms of classification and regression measures, such as accuracy, precision, recall, specificity, F1-score, AUC-ROC, and error metrics (when possible) (MAE and RMSE). ML components can also be evaluated in terms of interpretability.

IoMT components are evaluated with regard to sensing and communication reliability, such as latency, packet loss, signal-to-noise ratio, sampling resolution, bandwidth consumption, energy consumption, and thermal safety to achieve the continuous and safe monitoring of the neonatal condition. Blockchain elements are evaluated based on the measures of transaction latency, throughput, consensus overhead, immutability, resistance to attacks, and effectiveness in access-control.

**Figure 4:**
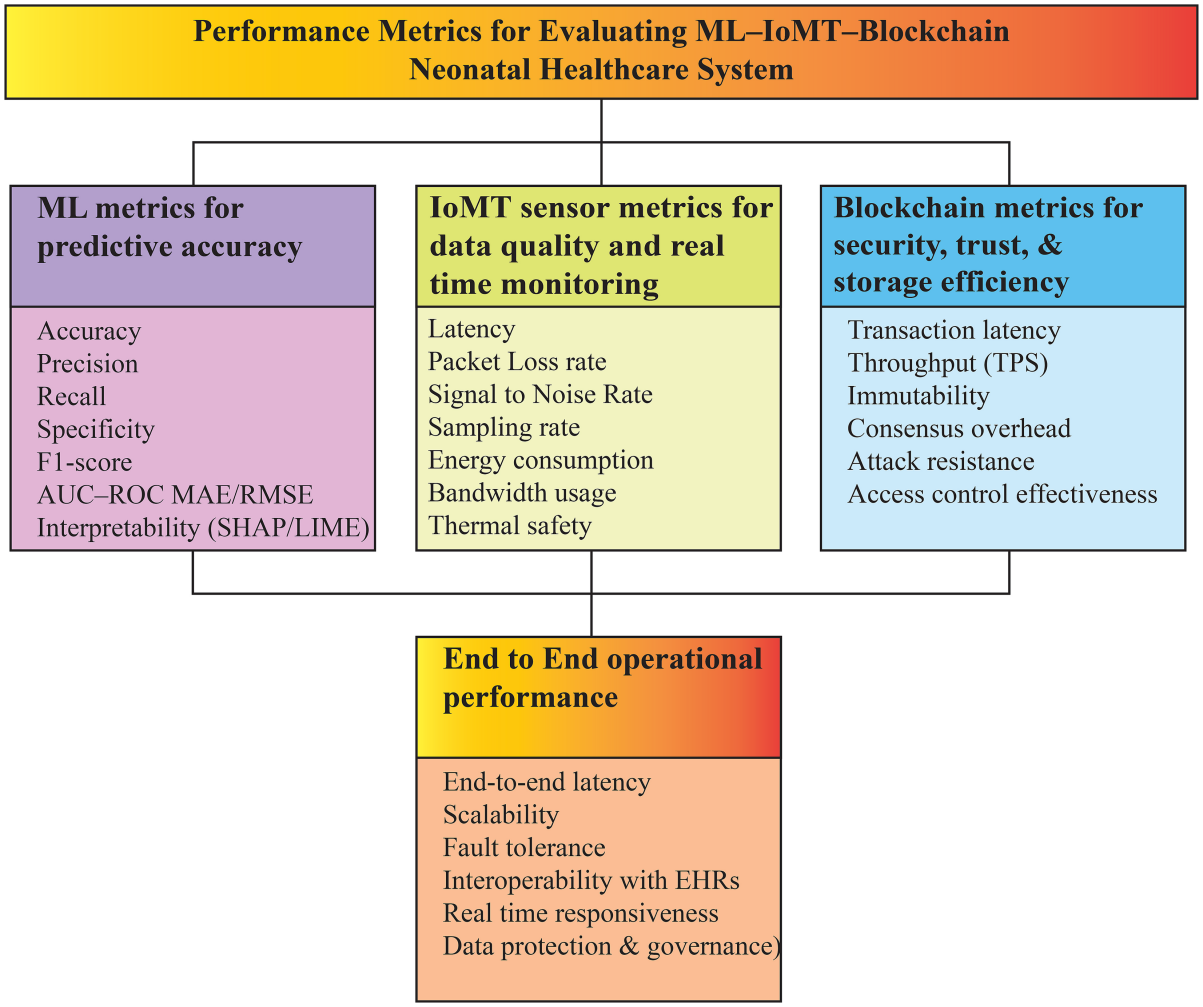
Unified evaluation and benchmarking framework for integrated ML–IoMT–Blockchain neonatal healthcare systems, illustrating predictive performance metrics, IoMT sensing reliability, blockchain security and governance measures, and end-to-end operational performance for NICU deployment.

Real-world applicability at the system level into NICU settings needs to take into account end-to-end latency, scalability, fault tolerance, electronic health records interoperability and responsiveness in real time. Clinical acceptability is also fostered by ethical and regulatory compliance such as auditability and compliance with GDPR/HIPAA principles. The combination of these measures offers a systematic benchmarking framework of the maturity and clinical preparedness of integrated ML-IoMT Blockchain neonatal healthcare systems as summarized in Figure 4.

### 4.5 Future directions for intelligent neonatal healthcare systems

The intersection of Machine Learning (ML) and the Internet of Medical Things (IoMT) and blockchain technologies is one of the most important directions of intelligent neonatal healthcare in the future. Their incorporation presents an opportunity to leave individual technological solutions and go to comprehensive, data-based, and reliable care structures that underpin individualized therapy and ongoing evaluation, as well as responsible and accountable clinical decision-making. Although the subject of collective use of ML, IoMT, and blockchain in the development of universal healthcare has been addressed in previous literature, including the work carried out by Babatunde et al., the use of each algorithm in neonatal care is relatively unexplored.

IoMT in the neonatal environment allows constant and real-time regulation of the vital physiological parameters and offers the opportunity to identify the occurrence of clinical degradation early. These high-resolution streams of data can be used through ML-based analytics to aid predictive risk management, personalized treatment strategies, and prompt clinical response. These capabilities are complemented by blockchain technologies which offer secure, decentralized data management, integrity, traceability, and transparent access control which is in accordance with parental consent and regulatory needs. Collectively, the technologies can facilitate NICU processes by automating the process of data collection, ensuring exchange of data is secure and accessible remotely, which will support coordination of care across institutions and geographical borders.

In spite of such opportunities, there are still severe technical, clinical, and governance challenges. These are, data imbalance and bias in neonatal datasets, resource limitations in NICU settings, limitations in interoperability across devices and platforms, and unethical and regulatory issues regarding data ownership and storage over time. The main future opportunities and challenges related to the integration of ML–IoMT–Blockchain presented in neonatal healthcare are outlined in Table 6, with directions on the research that is required to develop clinically validated, scalable, and trustworthy intelligent neonatal care systems. Moreover, more sophisticated anomaly-detection techniques can also be more applicable to neonatal surveillance settings which produce multimodal and time-changing sensor streams. Specifically, graph-based and contrastive learning methods can provide potential in the future to detect minor physiological or device-level abnormalities in the IoMT-based neonatal care system (Sefer, 2025).

**Table 6:** Future research opportunities and challenges associated with the integration of Machine Learning (ML), Internet of Medical Things (IoMT), and Blockchain technologies in neonatal healthcare systems.

Category	Future opportunities	Challenges
Machine Learning (ML)	Real-time prediction of life-threatening conditions in neonatal patients (e.g., sepsis; mortality risk) at high temporal resolution, which can be self-adaptive. Clinical complete modeling Multimodal neonatal analytics that combine physiological streams, imaging and longitudinal clinical records. Optimization of treatment by ML to individualize interventions in the NICU process (O’Sullivan et al., 2023; Narasimha Rao et al., 2024; Neal et al., 2025). Neonatal digital twins to allow risk reasoning and detection of abnormalities prior (Zaunseder et al., 2024).	Imbalance in classes and sampling bias in neonatal data; demographic and clinical heterogeneity which restrains the strength and external validity. Calculation and implementation load in resource-limited NICU settings (O’Sullivan et al., 2023; Narasimha Rao et al., 2024; Neal et al., 2025). Interpretable ML is needed to enable responsible decision-making and build clinician trust (Neal et al., 2025).
Internet of Medical Things (IoMT)	Non-invasive neonatal biosensors as contactless and wearable. Mist/edge computing Bedside/edge analytics with ultra-low latency. Tele-NICU in terms of the frequent follow-ups and monitoring (Mishra et al., 2023; Zhou et al., 2024). Cribs/Intelligent incubators and automated NICU (Krbec et al., 2024).	Minimization of devices, power consumption, and life cycle/maintenance. Is there an inter-manufacturer and inter-hospital barrier to interoperability? IoMT threats to safety include unauthorized access, spoofing, and tampering (Mishra et al., 2023; Zhou et al., 2024). Connectivity/bandwidth constraint in the rural and low-resource areas (Krbec et al., 2024).
Blockchain	Confirmed clinical workflow and record entries having unchangeable audit trails (Ullah et al., 2024). Access control and consent management were in line with parental consenting requirements (Kakarlapudi and Mahmoud, 2021). Safety-seeking data distribution among telehealth NICUs, hospitals, and researchers. Neonatal supply chain (e.g., medication, donor milk logistics) Traceability (Ng et al., 2021).	It is not ideal for usage in real-time NICU processes due to its high latency and resource overhead features. The performance limit of scaling under high rate IoMT streaming data (Ng et al., 2021). Demand the implementation of regulations and permissioned/hybrid blockchain architectures specific to that domain. Information sovereignty, ownership, and ethical limitations that must be regulated in operation (Kakarlapudi and Mahmoud, 2021; Ng et al., 2021).
Integrated ML–IoMT–Blockchain Ecosystem	Self-directed neonatal early-warning systems, sensing, prediction, and governance (Neal et al., 2025; Zhou et al., 2024; Ng et al., 2021). Cross-site data exchange, through secure registries that are standardized (Kakarlapudi and Mahmoud, 2021; Ng et al., 2021). Longitudinal learning of digital-twin-based pediatric care (Zaunseder et al., 2024). Real-time communication between NICUs, off-site hospitals and remote expertise (Zhou et al., 2024).	The lack of standardization between ML models, IoMT devices and distributed ledgers. Expensive cost of deployment and maintenance (Mishra et al., 2023). Training and trust in clinicians as a requirement to adoption (Zhou et al., 2024). Long-term storage, authorization, and data ownership remain persistent ethical concerns (Kakarlapudi and Mahmoud, 2021; Ng et al., 2021).

### 4.6 Research gaps and open challenges

The synthesis of research questions RQ1-7 suggests that machine learning, despite having reached methodological maturity in a number of fields of application, has little been translated into clinically deployable systems specific to neonatal applications. Most of the current literature in ML was based on the adult or mixed-patient population, and there is a lack of models that specifically address the specifics of the physiological peculiarities, developmental variability, and data limitations of neonates. The existing ML applications in the neonatal research are also concentrated focusing on a set of limited outcomes such as sepsis detection, mortality prediction, and neonatal jaundice, but such vital areas of the neonatal care as neurodevelopmental assessment, nutritional optimization, pain and stress quantification, and family-centered decision support are under researched.

Neonatal ML models are also constrained by methodological limitations to generalizability. In most cases, the basis of the vast majority of research is small, single-center, and highly class-imbalanced databases that have not been externally and multi-centreally validated. The performance measures that are used to model evaluation tend to be over-precise and over-AUC, and under-calibrated, under-clinical-useful, and under-vulcanized to distribution or model-interpretable changes. Moreover, the clinical efficacy and safety could not be evidenced much because the number of possible validation and real-time implementation studies was low within the NICU environment.

There is also a substantial gap in the use of IoMT in neonatal studies. Very few studies use high-resolution, multimodal sensor streams of data, including EEG, respiratory data or thermal imaging, even though they are applicable in neonatal monitoring. It is uncommon to use historical NICU data to learn from a longitudinal perspective, and system-level factors such as latency, bandwidth, power consumption and the feasibility of edge-computing are typically not considered in the design and testing of algorithms.

Blockchain structures have even more severe restrictions. Neonatal-specific Blockchain-IoMT solutions are few and most of the reported solutions are theoretical or at prototype stage, without empirical validation in actual NICU practices. The key factors of performance like end-to-end latency, ability to archive large volumes of sensor data at high frequencies, energy overhead, and the cost of storage immutability are rarely reported. Moreover, even though issues relating to parental consent management, interoperability of electronic health records, ownership of data and pediatric regulatory compliance is accepted, they are not often operationalized in deployed systems.

Altogether, comprehensive neonatal care systems based on entirely integrated ML, IoMT, and Blockchain are missing in the literature to a considerable extent. Current literature is usually limited to two layers of the technology stack (at best), and usually not within neonatal contexts. Less well-explored socio-technical aspects such as confidence and adoption by clinicians, parental trust, cross-institutional information sharing, and practicability on low-resource settings are additional impediments to real-life translation. Seeking to fill these gaps will necessitate the creation of more comprehensive neonatal datasets, viable and comprehensible ML systems, lifelike IoMT implementation research and mechanistically tested blockchain-based governance systems to enable the upcoming generation of smart, safe, and fair neonatal healthcare environments.

## 5 Conclusion

The reviewed article is a systematic synthesis of the existing evidence on neonatal care applications facilitated by the combined application of Machine Learning (ML), the Internet of Medical Things (IoMT), and Blockchain technologies based on the PRISMA-directed selection procedure. Out of a total of 122 records, 76 studies could be included based on the specified inclusion criteria and offered adequate information to answer the formulated research questions. It has been found that the most developed ML-based methods in neonatal care are related to predictive analytics, early disease detection, mortality risk estimation, and clinical decision support. Continuous and real-time monitoring of physiological processes, timely clinical intervention, and access control and integrity of data are the main concerns that IoMT technologies can serve, whereas blockchain-based solutions are the initial solutions to the challenges of neonatal healthcare that require data security, integrity, and trust among healthcare processes.

Collectively, ML, IoMT and blockchain represent a technological trio, which can help the emergence of intelligent neonatal healthcare systems of the next generation. This review introduces the systematic mapping of the area of application, patterns, and practices of evaluation and maturity gaps through which researchers, system designers and clinicians can find a common point of reference in designing secure, scalable and clinically meaningful neonatal solutions. Unlike the previous reviews, which have focused on these technologies individually, the current study will take a more comprehensive approach, not only by noting the opportunities but also by outlining the shortcomings of implementing integrated ML–IoMT -blockchain ecosystems in the neonatal intensive care setting. By and large, the review is in compliance with the global health priorities stated as part of Sustainable Development Goal 3, which focuses on the importance of reliable and data-oriented digital technologies to enhance the health outcomes and well-being of new-borns.

## Data Availability

The original contributions presented in the study are included in the article/supplementary material, further inquiries can be directed to the corresponding author.
